# Essential Oil from *Croton blanchetianus* Leaves: Anticandidal Potential and Mechanisms of Action

**DOI:** 10.3390/jof8111147

**Published:** 2022-10-29

**Authors:** Ellen A. Malveira, Pedro F. N. Souza, Nilton A. S. Neto, Tawanny K. B. Aguiar, Natanael S. Rodrigues, Carlos W. B. Henrique, Ayrles F. B. Silva, Leandro B. Lima, Cynthia C. Albuquerque, Cleverson D. T. Freitas

**Affiliations:** 1 Department of Biochemistry and Molecular Biology, Federal University of Ceará, Fortaleza 60020-181, Brazil; 2 Drug Research and Development Center, Department of Physiology and Pharmacology, Federal University of Ceará, Fortaleza 60430-160, Brazil; 3 Department of Biological Sciences, Faculty of Exact and Natural Sciences, State University of Rio Grande do Norte, Mossoró 59650-000, Brazil; 4 Department of Chemistry, Faculty of Exact and Natural Sciences, State University of Rio Grande do Norte, Mossoró 59650-000, Brazil

**Keywords:** essential oil, GC-MS/MS, biotechnological potential, *Candida* genus, antibiofilm activity

## Abstract

Antimicrobial drugs are becoming ineffective given the resistance acquired by microorganisms. As such, it is imperative to seek new antimicrobial molecules that could provide a basis for the development of new drugs. Therefore, this work aimed to evaluate the antimicrobial potential and the mechanisms of action of the essential oil extracted from leaves of *Croton blanchetianus* (named *Cb*EO) on different fungi and bacteria of clinical importance in both planktonic and biofilm lifestyles. GC-MS/MS analysis revealed the presence of twenty-two different compounds in the *Cb*EO, which were identified using the Kovats retention index. Among these, the most abundant were amorphene (20.03%), spathulenol (5%), bicyclogermacrene (1.49%), caryophyllene oxide (4.55%), and eucalyptol (5.62%). *Cb*OE (50 µg mL^−1^) barely inhibited the growth of *Bacillus subtilis* (23%), *Pseudomonas aeruginosa* (27%), and *Salmonella enterica* (28%), and no inhibition was obtained against *Enterobacter aerogenes* and *Klebsiella pneumoniae*. Additionally, no activity against bacterial biofilm was detected. In contrast, *Cb*EO was active against *Candida* species. *C. albicans* and *C. parapsilosis* were inhibited by 78 and 75%, respectively. The antibiofilm potential also was favorable against *C. albicans* and *C. parapsilosis*, inhibiting 44 and 74% of biofilm formation and reducing around 41 and 27% of the preformed biofilm, respectively. *Cb*OE caused membrane damage and pore formation, overproduction of ROS, and apoptosis on *C. albicans* and *C. parapsilosis* cells, as well as not inducing hemolysis in human red cells. The results obtained in this work raise the possibility of using the essential oil of *C. blanchetianus* leaves as an alternative to fight infections caused by *C. albicans* and *C. parapsilosis*.

## 1. Introduction

The misuse of antimicrobial drugs has dramatically intensified, generating a huge concern in health systems because of the emergence of multidrug-resistant pathogens, affecting around 5 million people worldwide [1]. Such conditions have driven humanity to an era referred to as post-antibiotic, making infections stronger and antibiotics useless [2]. For instance, infections caused by drug-resistant human pathogenic yeasts from the *Candida* genus, which affects healthy and immunocompromised people, are hard to treat. These infections range from superficial candidiasis localized on the skin to systemic and invasive bloodstream infections [3,4].

In addition, the formation of biofilms is another factor that enhance resistance of microorganisms to drugs [5]. Biofilm cells show differences in morphology, physiology, and gene expression compared to the planktonic form [6]. Moreover, they can be resistant to UV exposure, phagocytosis, and dehydration [7]. Thereby, drug-resistant microorganisms and their biofilms are problems that requires urgent attention and efforts to find new molecules to fight them back.

One of the alternatives for minimizing these problems is the search for new natural molecules, such as secondary metabolites produced by plants, which can inhibit the growth of resistant pathogens [8]. The Brazilian biomes are rich in many medicinal plants acting as biological sources for new molecules with therapeutic potential. Aromatic plants produce essential oils (EOs), defined as volatile and with strong odor characteristic, can be extracted from the leaves, barks, seeds, and fruits. In addition, some plant essential oils have been used to overcome microbial resistance [9,10]. 

**Figure 1:**
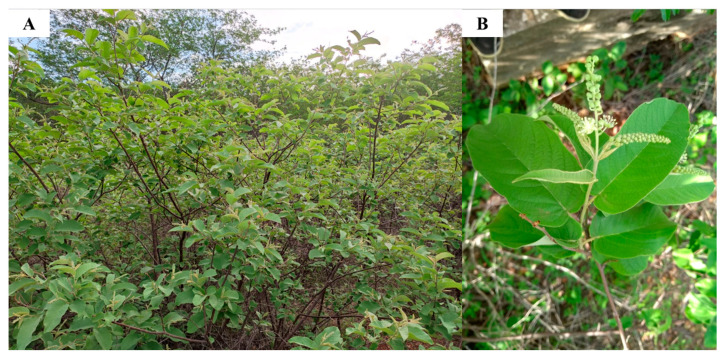
*C. blanchetianus* in the field. (**A**) Many *C. blanchetianus* trees and (**B**) *C. blanchetianus* leaves used in the oil extraction.

*C. blanchetianus* (Figure 1) Baill is an oil-producing plant belonging to the Euphorbiaceae family that have plants with several medicinal properties [11]. It has antibacterial, anti-inflammatory, and gastroprotective actions [9,10]. For instance, Melo et al. [9] reported that the essential oil from *C. blanchetianus* has a potent antibacterial activity against *Aeromonas hydrophila*, *Listeria monocytogenes*, and *Salmonella enteritidis* [9]. Additionally, oil of *C. blanchetianus* applied on meat was shown to prevent the growth of the foodborne pathogen *S. enteritidis*, thus showing the potential application of the oil in industry. Works with the essential oil from *C. blanchetianus* on planktonic and biofilm cells of bacteria and fungi of clinical importance and its respective mechanisms of action are still scarce. Based on the application of essential oil from *C. blanchetianus*, we hypothesized that the oil could have application against human pathogens. Therefore, this work aims to characterize and evaluate the antimicrobial and antibiofilm potential of the essential oil from *C. blanchetianus* leaves (*Cb*EO), as well as its mechanisms of action.

## 2. Results and Discussion

### 2.1. GC-MS/MS Analysis

Gas chromatography coupled with mass spectrometer (GC-MS/MS) revealed the presence of twenty-two different compounds in *Cb*EO (Table 1). Among these, the most abundant were amorphene (20.03%), spathulenol (5.81%), bicyclogermacrene (1.49%), caryophyllene oxide (4.55%), and eucalyptol (5.62%). Almost all the compounds found in *Cb*EO have been reported to present some biological activity. Although amorphene is the most abundant compound in the *Cb*EO (Table 1), to date, it has not been related to antimicrobial or antifungal activity. Usually, the biological activities attributed to amorphene are antioxidant and anti-leishmanial activity [12,13,14]. However, other abundant compounds have been associated with antimicrobial activity. For instance, spathulenol displays antifungal and antibacterial activities [12], bicyclogermacrene possesses antifungal and antioxidant activities [13], caryophyllene oxide has been reported to possess antioxidant, anticancer, and antimicrobial properties [14], and eucalyptol presents anticandidal activity [15].

In addition to these most abundant compounds, other components (Table 1) detected in *Cb*EO have also been reported with respect to their antimicrobial activity, such as limonene, borneol, α-terpineol, and sativene, presenting, respectively, anticandidal, antibacterial, and antifungal activities [15,16,17,18,19,20]. Furthermore, proteomic analysis of *C. albicans* cells treated with limonene revealed an up-accumulation of proteins involved with oxidative stress, DNA damage, nucleolar stress, and apoptosis [16].

### 2.2. Antimicrobial Activity

*Cb*OE was tested against several human-pathogenic bacteria and yeasts (Table 2). The antibacterial activity of *Cb*OE barely inhibited the growth of *Bacillus subtilis* (23%), *Pseudomonas aeruginosa* (27%), and *Salmonella enterica* (28%), and no inhibition was observed against *Enterobacter aerogenes* and *Klebsiella pneumoniae*, even using the highest concentration tested (50 µg mL^−1^). Likewise, no activity against the bacterial biofilms was detected at 50 µg mL^−1^ (Table 2). In a previous study [21], it was shown that the aromadendrene present in the EO of *Eucalyptus globulus* has remarkable activity on strains of methicillin-resistant *S. aureus* (MRSA), vancomycin-resistant *Enterococcus faecalis* (VRE), and *Acinetobacter baumanii*. However, the inefficiency of *Cb*EO against bacteria can be explained by the low concentration of potent antibacterial molecules, such as borneol, α-terpineol, and sativene found in other essential oils [15,16,17,18,19,20].

Regarding anticandidal activity, *Cb*EO presented better results against *C. albicans* and *C. parapsilosis*, with inhibition of 78 and 75%, respectively, at 50 µg mL^−1^ (Table 2). Otherwise, *C. krusei* was barely susceptible (9.5% of inhibition) and *C. tropicalis* was not affected by treatment with *Cb*EO even at the highest concentration tested (50 µg mL^−1^). The antibiofilm potential of *Cb*OE (50 µg mL^−1^) was also favorable against *C. albicans* and *C. parapsilosis,* inhibiting 44 and 74% of biofilm formation and reducing preformed biofilm by 41 and 27%, respectively (Table 2). These results are in agreement with higher concentrations of spathulenol, bicyclogermacrene, caryophyllene oxide, and eucalyptol, which have been described as potent antifungal and anticandidal compounds [12,13,14,15]. Keymaram et al. [22] showed in their study that eucalyptol possesses antibiofilm activity against *C. albicans* at concentrations ranging from 125 to 8000 μg mL^−1^. Although detected at a low concentration in *Cb*EO, limonene could also contribute to anticandidal activity, specifically against *C. albicans,* since this compound has already shown anticandidal activity [16]. Al-Ghanayem [23] showed that lemongrass leaf oil (*Cymbopogon flexuosus*) was active against *C. albicans* planktonic cells, and complete biofilm reduction could be observed at 0.5 μL mL^−1^ and 30% at a concentration of 0.03 μL mL^−1^.

In another study, the Pistachio hull essential oil completely inhibited the growth of *C. parapsilosis* at a concentration of 2.50 mg mL^−1^ and slightly inhibited *C. glabrata* and *C. albicans* at the same concentration [24]. It seems that the results presented by *Cb*EO are exciting when compared to those above. Compared to D’Arrigo et al. [24], EO was effective against *C. parapsilosis* at a concentration 50 times lower than that required for essential oil from Pistachio hull. In addition, *Cb*EO was also effective against *C. parapsilosis* biofilm (Table 2). Natural products are considered strong inhibitors when they have an MIC_50_ of up to 0.5 mg mL^−1^, moderate inhibitors between 0.6 and 1.5, and weak inhibitors when this value is higher than 1.6 mg mL^−1^ (Madeira et al., 2016). Corroborating this work, *Cb*EO is considered a strong inhibitor of *C. albicans* and *C. parapsilosis*, since it shows activity at 0.05 mg mL^−1^ (Table 2).

*C. citrate* oil with silicone rubber coating inhibited *C. tropicalis* biofilm formation by between 45 and 76% [25]. Here, our results revealed that *Cb*EO was not effective against *C. tropicalis.* This, indeed, is not a negative result. However, it is an interesting result because shows selectivity. The literature has already discussed that different essential oils may present distinct biological activities based on their composition [23]. The activity of *Cb*EO against *C. albicans* and *C. parapsilosis* biofilms has a great potential to drive further studies to develop new drugs against biofilms. As already mentioned, biofilm formation is one of the mechanisms of fungal resistance occasioned by *Candida* species that may be associated with medical devices such as cardiovascular, venous, and urinary catheters [26].

### 2.3. Mechanisms of Action

#### 2.3.1. Membrane Damage and Pore Formation

There are other studies evaluating the anticandidal activity of essential oils from many plants. However, few have shown the mechanism of action. Thakre et al. [16] revealed by proteomic analysis the up accumulation of proteins related to damage to DNA and nucleolar region, suggesting induction of apoptosis in *C. albicans* cells treated with limonene. Recently, Yu et al. [19] reported that limonene induced mitochondrial membrane depolarization and cell membrane pore formation.

**Figure 2:**
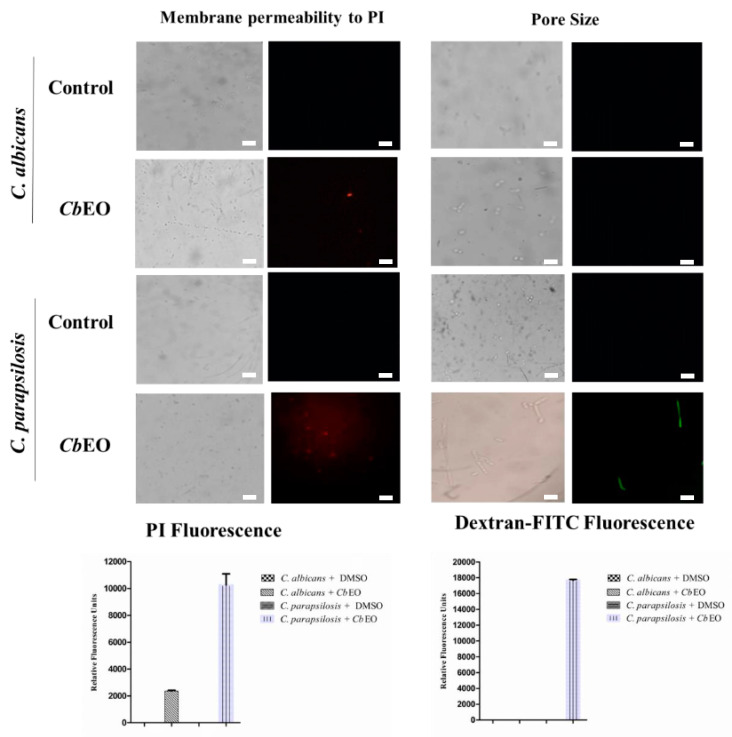
Fluorescence images showing membrane damage and pore size on planktonic cells of *C. albicans* and *C. parapsilosis*. The membrane damage was assayed by propidium iodide (PI) uptake and pore size by using a 6 kDa dextran-FITC. The control was 5% DMSO. Bars indicates 100 µm.

**Figure 3:**
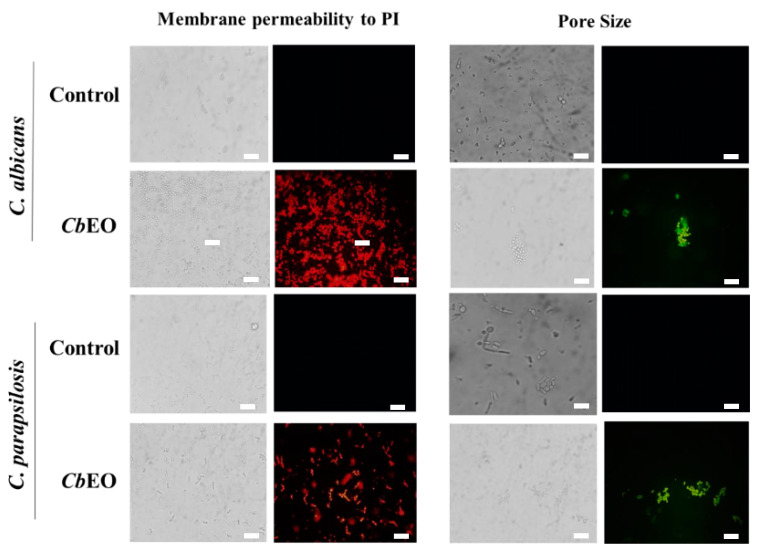
Fluorescence images showing membrane damage and pore size on biofilm cells of *C. albicans* and *C. parapsilosis*. The membrane damage was assayed by propidium iodide (PI) uptake and pore size by using a 6 kDa FITC-Dextran. The control was 5% DMSO. Bars indicates 100 µm.

Here, we provide evidence about how the *Cb*EO displays anticandidal activity. The propidium iodide (PI) uptake assay was employed to evaluate the membrane damage in *C. albicans* and *C. parapsilosis* planktonic and biofilm cells. PI interacts with DNA, releasing red fluorescence. However, it can only move through damaged membrane, thus indicating pore formation. Therefore, healthy membranes do not allow the passage of PI through them. The data obtained regarding the planktonic cells showed no fluorescence of PI on control (5% DMSO) cells (Figure 2). In contrast, *C. albicans* and *C. parapsilosis* cells treated with *Cb*EO showed red fluorescence, indicating membrane damage (Figure 2). The fluorescence was quantified and showed that *C. parapsilosis* cells were more susceptible to membrane damage caused by *Cb*EO than *C. albicans* (Figure 2). Similarly, *Cb*EO was also able to induce membrane permeabilization in both *C. albicans* and *C. parapsilosis* biofilms (Figure 3). Indeed, *Cb*EO was more efficient against *C. albicans* biofilms than in planktonic cells (Figure 3). The membrane damage induced by essential oils from plants has already been discussed [27]. However, there are no studies indicating the size of the pore formed.

Although PI indicates damage to the cell membrane, it does not give any information about pore size. Thus, new assays were performed using a fluorophore with a size of 6 kDa (Dextran-FITC). Only *C. parapsilosis* planktonic cells showed green fluorescence, indicating that *Cb*EO allowed the movement of 6 kDa FITC-dextran (Figure 3). In contrast, green fluorescence was observed in biofilm cells of both *C. albicans* and *C. parapsilosis* (Figure 2). Etxaniz et al. [28] revealed that in some cases, the cell membrane can recover from the formation of a pore. For example, the pores revealed by PI have a size of around 0.1 nm, making it possible for the cell to recover. However, pores revealed by FITC-Dextran have a size of 1.0 nm, and are thus classified as large pores, making it quite difficult for the cells to recover [28], since there may be extravasation of various cellular molecules, membrane depolarization, and induction of apoptosis, as reported by Thakre et al. [16].

The *Zanthoxylum schinifolium* essential oil had activity on membrane permeabilization against the fungus *Malassezia restricta* [29]. Terpenoids have already been reported to alter membrane fluidity and modulation of proteins linked to signaling and transport [30]. In a previous study [31], it was shown that terpinen-4-ol can impair the integrity and physiology of fungal cells by inducing membrane loss.

#### 2.3.2. Overproduction of Reactive Oxygen Species (ROS)

**Figure 4:**
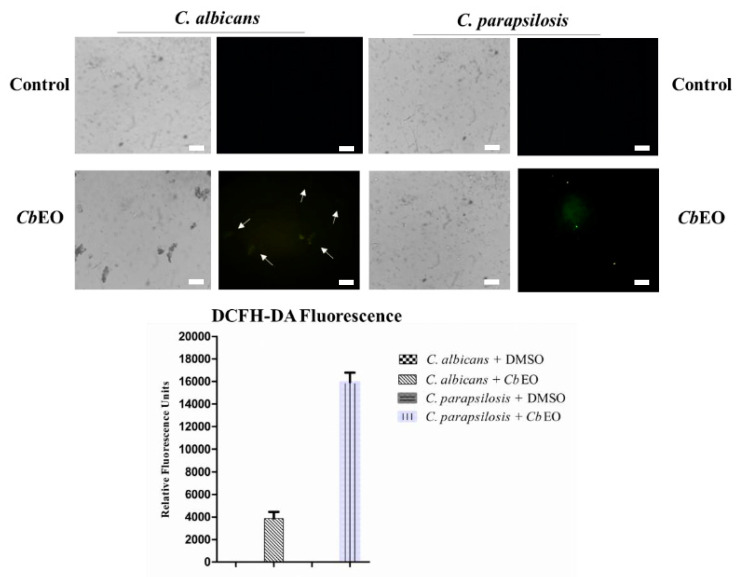
Fluorescence images showing ROS overproduction (Green Fluorescence) by planktonic cells of *C. albicans* and *C. parapsilosis*. The control was 5% DMSO. White arrows indicate cell with green fluorescence. Bars indicates 100 µm.

**Figure 5:**
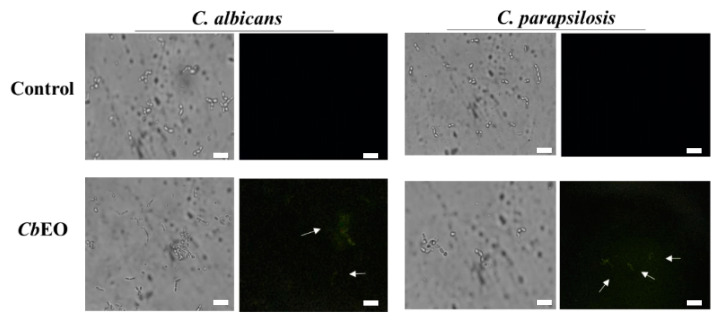
Fluorescence images showing ROS overproduction (Green Fluorescence) by biofilm cells of *C. albicans* and *C. parapsilosis*. The control was 5% DMSO. White arrows indicate cells with green fluorescence. Bars indicates 100 µm.

To further explore the mechanism of action, the ROS overproduction induced by *Cb*EO in both *C. albicans* and *C. parapsilosis* cells was evaluated. The *Cb*EO (50 µg mL^−1^) induced ROS overproduction (Figure 4) in planktonic cells of both yeasts (green fluorescence). However, there was a difference in the intensity of ROS produced by both species. The quantification of fluorescence revealed a higher amount of ROS produced by *C. parapsilosis* (Figure 4). As expected, the controls did not have an overproduction of ROS. *Cb*EO also induced ROS overproduction in biofilms of both *C. parapsilosis* and *C. albicans* (Figure 5—white arrows), albeit to a lesser extent.

Ding et al. [32] reported that ROS levels increased in *C. albicans* cells after incubation with a quinoline compound. Yang et al. [33] showed that lavender essential oil induced oxidative stress in *K. pneumonia*, leading to membrane permeabilization and cell death. Indeed, the high level of ROS could be lethal to cells because it can lead to damage to critical molecules such as DNA, protein, and lipids [34]. Looking the composition of lavender essential [33], the presence of limonene, borneol, and caryophyllene was also present in *Cb*EO. These compounds, present in both oils, could be involved in ROS overproduction in microbial cells. ROS is essential to biofilm biogenesis, development, and formation, as well as the genetic variability of cells [35]. However, the line between benefits and lethal effects is thin and easy to cross. A slight imbalance of ROS levels can lead to its accumulation, which is lethal, because it inactivates vital molecules such as carbohydrates, nucleic acids, proteins, and lipids, triggering programmed cell death [36].

#### 2.3.3. Caspase 3/7-Mediated Apoptosis

**Figure 6:**
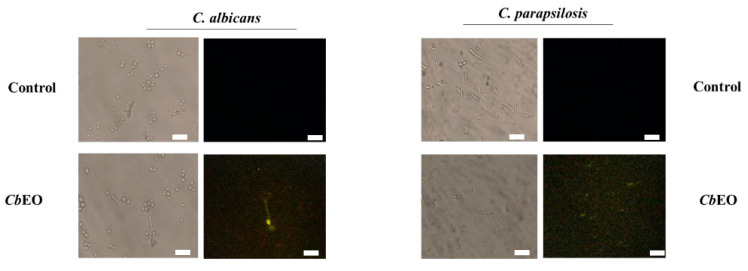
Evaluation of apoptosis in *C. albicans* and *C. parapsilosis* strains under the action of *Cb*EO at the concentration of 50 μg mL^−1^. Bars indicates 100 µm.

**Figure 7:**
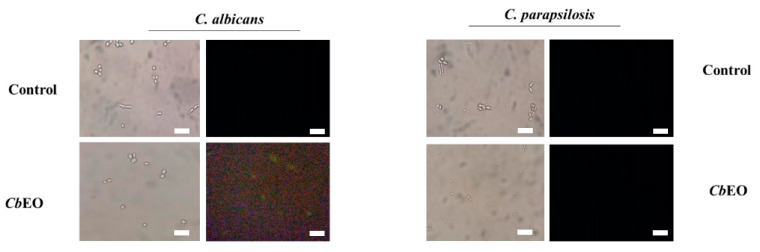
Evaluation of apoptosis in *C. albicans* and *C. parapsilosis* biofilms under the action of *Cb*EO at the concentration of 50 μg mL^−1^. Bars indicates 100 µm.

*Cb*EO induced caspase 3/7-mediated apoptosis in planktonic cells of *C. albicans* and *C. parapsilosis* and weakly in biofilms of *C. albicans* only (Figure 6 and Figure 7). The exact mechanism of how *Cb*EO induced apoptosis in the *C. parapsilosis* cells has not yet been elucidated. Caspase-3/7 starts apoptotic DNA fragmentation by activating of a protein called DNA fragmentation factor-45 (DFF45) and an inhibitor of caspase-activated DNase (ICAD). Thakre et al. [16] revealed by proteomic analysis that the treatment with limonene increase the accumulation of proteins involved with DNA damage and apoptosis in *C. albicans* cells. However, as happens here, the authors did not understand the mechanism involved.

#### 2.3.4. Scanning Electron Microscopy (SEM)

**Figure 8:**
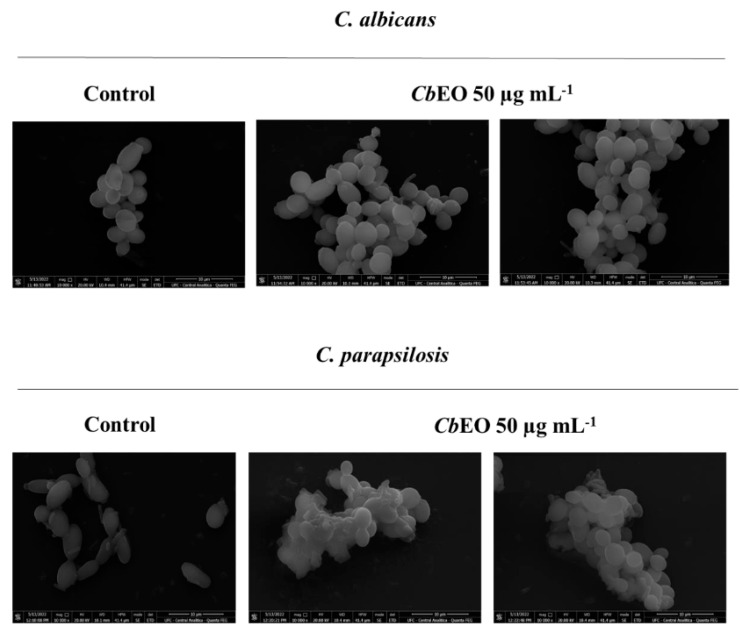
Scanning electron microscopy of *C. albicans* and *C. parapsilosis* cells after the action of *Cb*EO at the concentration of 50 μg mL^−1^. Magnification 10,000×.

SEM was also used to evaluate the damage on *C. albicans* and *C. parapsilosis* cells caused (Figure 8) after treatment with *Cb*EO (50 µg mL^−1^). *Cb*EO caused changes in the morphology of cells, scars, roughness, and depletions leading to loss of internal content corroborating with the data from fluorescent microscopy. In contrast, control cells did not present any damage (Figure 8). The SEM analysis provided important results, because there were not many works evaluating the cellular damage caused by EOs in yeast cells.

#### 2.3.5. Hemolytic Activity

To be considered as potential molecules for the development of new drugs, the candidate should not present any or very low toxicity to hosts [37]. Based on the data obtained, *Cb*EO does not show toxicity to blood types A, B and O, even at the highest concentration tested (250 µg mL^−1^), when compared to the control (DMSO 5%) (Table 3).

## 3. Conclusions

This study highlights relevant results about the potential of *Cb*EO as a source of anticandida molecules. These data are essential for understanding the possible application of *Cb*EO in treating infections caused by *C. albicans* and *C. parapsilosis*. A future perspective is essential to notice that *Cb*EO is not toxic to human red blood cells, which, together with anticandidal activity, opens up great prospects for its application in the future. The main conclusion is that *Cb*EO possesses potent anticandida activity in both planktonic and biofilm lifestyles and presents no danger to human red blood cells.

## 4. Experimental Section

### 4.1. Biological Material

The leaves of *Croton blanchethianus* Baill. were collected in the city of Mossoró, Rio Grande do Norte, Brazil (latitude: −5.201324, longitude: −37.320572). Regarding microorganisms, the yeasts *C. albicans* (ATCC 10231), *C. krusei* (ATCC 6258), *C. parapsilosis* (ATCC 22019), *Cryptococcus neoformans* (ATCC 32045) and *C. tropicalis* (Clinical isolate) and the bacteria *B. subtilis* (ATCC 6633), *E. aerogenes* (ATCC 13048), *K. pneumoniae* (ATCC 10031), *P. aeruginosa* (ATCC 25619), and *S. enterica* (ATCC 14028) were obtained of the Laboratory of Plant Toxins of the Federal University of Ceará, Brazil.

### 4.2. Oil Extraction

The original equipment was provided by the Laboratory of Plant Physiology and Biochemistry (LFBP). For the extraction process, the methodology proposed by Oliveira et al. was followed [38]. We decided to use the leaves because they have the largest amount of glandular trichomes, for the storage and synthesis of bioactive metabolites such as the terpenoids and flavonoids that are present. The essential oil from the leaves of *C. blanchethianus* (*Cb*EO) was extracted by the hydrodistillation method using a Clevenger apparatus. In the Clevenger apparatus, samples are diluted in water, which is boiled to evaporate volatile components in the steam. In this way, two layers (aqueous and oil-rich) are obtained, and oil is separated using separating funnels. For extraction, the leaves were weighed (586 g) on a precision balance and then placed in a volumetric flask containing 2 L of distilled water. The flask was attached and heated on a heating mantle. After boiling, the steam generated was condensed, and oil and water were collected in a Dean–Stark apparatus. As they are immiscible, two phases were formed, and it was possible to separate oil and water. The oil was extracted for a period of 2 h, controlling the temperature to approximately 100 °C. The essential oil was dehydrated 6 g of anhydrous sodium sulfate (PM:142.04) and 10 mL of ethyl ether, resulting 8 mL of oil, which was stored under refrigeration (4 °C).

### 4.3. Characterization of CbEO by GC-MS/MS Analysis

The chemical composition of *Cb*EO was examined by gas chromatography coupled with mass spectrometry (GC-MS/MS) (Shimadzu GCMS-QP2010 SE, Kyoto, Japan), which was equipped with an Rtx^®^-5MS capillary column (30 m × 0.25 mm × 0.25 μm). The operating conditions of the GC-MS/MS were optimized as follows: 70 eV, carrier gas (He), flow rate of 1.7 mL.min^−1^, and pressure 53.5 KPa. The temperatures of the injector and the interface of the detector were 25 °C and 230 °C, respectively. The oven temperature program was 100 °C for 3 min, and then 310 °C at a heating rate of 3.5 °C/min and maintained at 310 °C for 5 min. The identification of the constituents of the essential oils was investigated by comparing the mass spectra and Kovats index values (IK) with those of the library search references.

### 4.4. Antimicrobial and Antibiofilm Activities

The anticandidal activity was evaluated using the microdilution method described by the Clinical and Laboratory Standards Institute [39] with some modifications. To evaluate cell growth inhibition, an aliquot (50 µL) of yeast cell suspensions (0.5–2.5 × 10^6^ CFU mL^−1^) in Saboraud liquid medium was mixed in 96-well plates with 50 µL of *Cb*EO (ranging from 50 to 0.008 µg mL^−1^, diluted in 5% DMSO). 

The antibacterial activity was determined according to the method described by Oliveira et al. [40] with modifications. The experimental assay was similar to the previous one, with the bacterial cells being cultivated in Mueller–Hinton Broth medium. The positive controls were nystatin and ciprofloxacin and the negative control was 5% DMSO. After 24 h at 37 °C, the cell growth was measured using a microplate reader at 600 nm (Epoch, BioTek Instruments Inc., Winooski, VT, USA). Each experiment was performed three times, with three replicates per treatment. 

The antibiofilm assays were performed according to the method described by Dias et al. [41]. The same controls and concentrations of *Cb*EO used in planktonic cells were applied in the biofilm assays. After 24 h of incubation, the supernatant was removed from the wells, followed by three washes with 0.15 M NaCl, and then 200 μL of methyl alcohol was added to each well for 15 min to allow fixation of the adhered cells to occur. Then, 200 μL of 0.1% crystal violet was added for another 15 min. To dissolve the dye attached to the biofilm, 200 μL of 33% acetic acid was added and left on the plate for reading the absorbance at 590 nm using a plate reader. 

### 4.5. Mechanisms of Action

#### 4.5.1. Membrane Damage

For cell membrane integrity assay, 50 µL of *C. albicans* or *C. parapsilosis* cell suspension (2.5 × 10^3^ CFUmL^−1^, in Saboraud liquid medium) was mixed with 50 µL of *Cb*EO (50 µg mL^−1^) and incubated for 24 h at 37 °C. Then, the samples were centrifuged at 5000× *g* for 5 min at 4 °C, and the cells were washed three times with 100 µL of 0.15 M NaCl. Subsequently, 50 µL of NaCl and 3 µL of 1 mM propidium iodide (PI) were added and the mixture was incubated for 30 min in the dark at 37 °C. Afterward, the samples were washed twice with 0.15 M NaCl to remove excess PI and the cells were resuspended in 50 µL of 0.15 M NaCl to be analyzed on a fluorescence microscope (Olympus System BX 41, Tokyo, Japan) with an excitation wavelength of 535 nm and emission wavelength of 617 nm.

Additionally, the samples were treated similarly to the previous analysis and incubated with 3 µL of 1 mM 6 kDa FITC-Dextran (Sigma Aldrich, Sao Paulo, Brazil) in the dark for 30 min, according to Oliveira et al. [40]. The result was observed under a fluorescence microscope (Olympus System BX60) with an excitation wavelength of 488 nm and an emission wavelength of 525 nm.

#### 4.5.2. Induction of Reactive Oxygen Species (ROS)

To perform ROS overproduction, the methodology described by Dikalov and Harrison [42] was followed. The experimental design was similar to the previous assays of antimicrobial activity. First 50 µL of *C. albicans* or *C. parapsilosis* cell suspension (2.5 × 10^6^ CFU mL^−1^, in Saboraud liquid medium) was mixed with 50 µL of *Cb*EO (50 µg mL^−1^) and incubated for 24 h at 37 °C. The samples were washed three times with 0.15 M NaCl solution and incubated with 50 µL of 0.2 M DCFH-DA for 20 min in the dark. A fluorescence microscope (Olympus System BX60) was used with an excitation wavelength of 485 nm and an emission wavelength of 538 nm.

#### 4.5.3. Induction of Apoptosis

The caspase activity was measured after cell incubation for 24 h, in the presence and absence of essential oil, according to the manufacturer’s instructions. The cells were treated as above and then incubated using 3 μL of 2 mM CellEvent^®^ reagent (ThermoFisher, São Paulo, SP, Brazil) for 30 min in the dark. Then, cells were washed and centrifuged as described above. Finally, the cells were observed under a fluorescence microscope (Olympus System BX60) at an excitation wavelength of 342 nm and an emission wavelength of 441 nm.

### 4.6. Scanning Electron Microscopy (SEM)

For SEM analysis, the samples were mixed under the same conditions described previously, and then glutaraldehyde in 0.15 M sodium phosphate buffer pH 7.2 was added for 16 h at 25 °C for fixation. After that, each sample was washed three times with 0.15 M sodium phosphate buffer pH 7.2. For dehydration, it was washed with ethanol at different concentrations (30, 50, 70, and 100%) leaving it for 10 min, except for the last concentration, which was dehydrated twice for 10 min. Subsequently, 50% hexamethyldisilane (HMDS, Sigma, St. Louis, MI, USA) was diluted in ethanol for 10 min and then 100% HMDS was added. A 15 µL aliquot was added to coverslips and made it possible to dry at room temperature. For observation of the cells, they were coated with gold on aluminum surfaces and observed under a scanning electron microscope (Everhart–Thornley) [43].

### 4.7. Hemolytic Activity

The hemolytic activity of *Cb*EO was tested against red blood cells (A, B and O^+^) according to Oliveira et al. [40]. Blood types were provided by the Center for Hematology and Hemotherapy of Ceará (Brazil). Cells were centrifuged at 5000× *g* for 5 min at 4 °C, and dissolved in 0.15 M NaCl. Six washes were performed, and then the bloods were diluted to 2.5% in 0.15 M NaCl. Subsequently, an aliquot of 300 μL of each blood type was incubated with 300 μL of *Cb*OE at concentrations ranging from 50 to 12.5 μg mL^−1^, while the negative control contained 5% DMSO and the positive control 0.1% (*v*/*v*) Triton X-100. Then, the samples were incubated for 30 min at 37 °C, followed by centrifugation (5000× *g* for 5 min at 4 °C). After that, the supernatants were collected and transferred to 96-well plates. Hemolysis (%) was calculated by measuring the absorbance of the supernatant at 414 nm using a microplate reader.

### 4.8. Statistical Analysis

All tests were performed in three biologically independent experiments. The difference between the means of the triplicates was verified by applying the ANOVA test followed by the Tukey method using the GraphPad Prism program version 5.01 (GraphPad Software company, Santa Clara, CA, USA). Values of *p* < 0.05 were considered statistically significant.

## Figures and Tables

**Table 1:** Compounds identified in essential oil from *C. blanchetianus* leaves (*Cb*EO).

Compound	Retention Time	Area (%)
limonene	6.02	0.61
eucalyptol	6.14	5.62
borneol	9.84	0.64
terpinen-4-ol	10.13	1.32
α-terpineol	10.52	1.16
myrtenol	10.70	0.70
δ-elemene	14.61	0.46
α-ylangene	15.67	0.51
β-bourbonene	15.92	0.95
sativene	16.15	1.99
E-caryophyllene	16.86	1.95
aromadendrene	17.37	0.56
6,9-guaiadiene	17.47	1.31
α-humulene	17.75	0.73
alloaromadendrene	17.95	1.10
germacrene D	18.47	0.57
γ-himachalene	18.61	0.70
bicyclogermacrene	18.90	0.57
δ-amorphene	19.54	5.92
spathulenol	21.08	1.49
caryophylleneoxide	21.16	20.03
epi- α-muurolol	22.79	5.81
		4.55
		2.98

**Table 2:** Evaluation of *Cb*EO on planktonic cells and biofilm formation of some microorganisms.

*Cb*EO (50 µg mL^−1^)				
**Antifungal activity**				
	Inhibition of planktonic cells growth (%)	Inhibition of biofilm formation (%)	Biomass reduction of preformed biofilm (%)	Nystatin
*C. albicans* ATCC 10231	78.55 ± 0.3	44.12 ± 0.2	41.12 ± 0.4	85.75 ± 0.1
*C. krusei* ATCC 6258	9.58 ± 0.5	00.00	00.00	75.00 ± 0.6
*C. parapsilosis* ATCC 22019	75.94 ± 0.1	74.13 ± 0.7	27.75 ± 0.8	86.66 ± 0.3
*C. tropicalis* clinical isolate	00.00	00.00	00.00	93.85 ± 0.9
*C. neoformans* ATCC 32045	00.00	00.00	00.00	75.02 ± 0.5
**Antibacterial activity**				**Ciprofloxacin**
*B. subtilis* ATCC 6633	23.00 ± 0.3	00.00	00.00	85.42 ± 0.4
*E. aerogenes* ATCC 13048	00.00	00.00	00.00	84.23 ± 0.4
*K. pneumoniae* ATCC 10031	00.00	00.00	00.00	81.68 ± 0.7
*P. aeruginosa* ATCC 25619	27.01 ± 0.7	00.00	00.00	87.39 ± 0.8
*S. enterica* ATCC 14028	28.12 ± 0.5	00.00	00.00	87.74 ± 0.2

**Table 3:** Hemolytic activity of *Cb*EO against different human red blood cells.

Blood Type			Hemolysis (%)				
	0.1% Triton	5% DMSO	*Cb*EO 250 µg mL^−1^	*Cb*EO 150 µg mL^−1^	*Cb*EO 100 µg mL^−1^	*Cb*EO 50 µg mL^−1^	*Cb*EO 25 µg mL^−1^
**Type A**	100	0	0	0	v	0	0
**Type B**	100	0	0	0	0	0	0
**Type O**	100	0	23	0	0	0	0

## Data Availability

The data supporting this study’s findings are available on request from the corresponding author.

## References

[1] Murray C.J., Ikuta K.S., Sharara F., Swetschinski L., Robles Aguilar G., Gray A., Han C., Bisignano C., Rao P., Wool E. (2022). Global Burden of Bacterial Antimicrobial Resistance in 2019: A Systematic Analysis. Lancet.

[2] WHO (2014). Antimicrobial Resistance.

[3] López-Martínez R. (2010). Candidosis, a New Challenge. Clin. Dermatol..

[4] Papon N., Courdavault V., Clastre M., Bennett R.J. (2013). Emerging and Emerged Pathogenic *Candida* Species: Beyond the *Candida albicans* Paradigm. PLoS Pathog..

[5] Do Vale J.P.C., Ribeiro L.H.d.F., de Vasconcelos M.A., Sá-Firmino N.C., Pereira A.L., do Nascimento M.F., Rodrigues T.H.S., da Silva P.T., de Sousa K.C., da Silva R.B. (2019). Chemical Composition, Antioxidant, Antimicrobial and Antibiofilm Activities of *Vitex gardneriana* Schauer Leaves’s Essential Oil. Microb. Pathog..

[6] Olivares E., Badel-Berchoux S., Provot C., Prévost G., Bernardi T., Jehl F. (2020). Clinical Impact of Antibiotics for the Treatment of *Pseudomonas aeruginosa* Biofilm Infections. Front. Microbiol..

[7] Gupta P., Sarkar S., Das B., Bhattacharjee S., Tribedi P. (2016). Biofilm, Pathogenesis and Prevention—A Journey to Break the Wall: A Review. Arch. Microbiol..

[8] Mathur S., Hoskins C. (2017). Drug Development: Lessons from Nature. Biomed. Rep..

[9] Melo G.F.d.A., da Costa A.C.V., Garino F., Medeiros R.S., Madruga M.S., Neto V.Q. (2013). The sensitivity of bacterial foodborne pathogens to *Croton blanchetianus* Baill essential oil. Braz. J. Microbiol..

[10] Aquino V.V.F., Costa J.G.M., Angélico E.C., Medeiros R.S., Lucena M.d.A., Rodrigues O.G. (2017). Metabólitos secundários e ação antioxidante de *Croton heliotripifolius* e *Croton blanchetianus*. Acta Bras..

[11] Freitas A.F.S., Costa W.K., Machado J.C.B., Ferreira M.R.A., Paiva P.M.G., Medeiros P.L., Soares L.A.L., Oliveira A.M., Napoleão T.H. (2020). Toxicity Assessment and Antinociceptive Activity of an Ethanolic Extract from *Croton blanchetianus* (Euphorbiaceae) Leaves. South Afr. J. Bot..

[12] Cazella L.N., Glamoclija J., Soković M., Gonçalves J.E., Linde G.A., Colauto N.B., Gazim Z.C. (2019). Antimicrobial Activity of Essential Oil of *Baccharis dracunculifolia* DC (Asteraceae) Aerial Parts at Flowering Period. Front. Plant Sci..

[13] Nogueira Sobrinho A.C., de Morais S.M., Marinho M.M., de Souza N.V., Lima D.M. (2021). Antiviral Activity on the Zika Virus and Larvicidal Activity on the *Aedes* Spp. of *Lippia alba* Essential Oil and β-Caryophyllene. Ind. Crops Prod..

[14] Dahham S., Tabana Y., Iqbal M., Ahamed M., Ezzat M., Majid A., Majid A. (2015). The Anticancer, Antioxidant and Antimicrobial Properties of the Sesquiterpene β-Caryophyllene from the Essential Oil of *Aquilaria crassna*. Molecules.

[15] Ivanov M., Kannan A., Stojković D.S., Glamočlija J., Calhelha R.C., Ferreira I.C.F.R., Sanglard D., Soković M. (2020). Flavones, Flavonols, and Glycosylated Derivatives—Impact on *Candida albicans* Growth and Virulence, Expression of CDR1 and ERG11, Cytotoxicity. Pharmaceuticals.

[16] Thakre A., Zore G., Kodgire S., Kazi R., Mulange S., Patil R., Shelar A., Santhakumari B., Kulkarni M., Kharat K. (2018). Limonene Inhibits *Candida albicans* Growth by Inducing Apoptosis. Med. Mycol..

[17] Ai Y., Fang F., Zhang L., Liao H. (2022). Antimicrobial Activity of Oregano Essential Oil and Resveratrol Emulsions Co-Encapsulated by Sodium Caseinate with Polysaccharides. Food Control.

[18] Guimarães A.C., Meireles L.M., Lemos M.F., Guimarães M.C.C., Endringer D.C., Fronza M., Scherer R. (2019). Antibacterial Activity of Terpenes and Terpenoids Present in Essential Oils. Molecules.

[19] Yu H., Ren X., Yang F., Xie Y., Guo Y., Cheng Y., Yao W. (2022). Antimicrobial and Anti-dust Mite Efficacy of Cinnamomum Camphora Chvar. Borneol Essential Oil Using Pilot-plant Neutral Cellulase-assisted Steam Distillation. Lett. Appl. Microbiol..

[20] Zhou H., Tao N., Jia L. (2014). Antifungal Activity of Citral, Octanal and α-Terpineol against *Geotrichum citri-aurantii*. Food Control.

[21] Mulyaningsih S., Sporer F., Zimmermann S., Reichling J., Wink M. (2010). Synergistic Properties of the Terpenoids Aromadendrene and 1,8-Cineole from the Essential Oil of *Eucalyptus globulus* against Antibiotic-Susceptible and Antibiotic-Resistant Pathogens. Phytomedicine.

[22] Keymaram M., Falahati M., Farahyar S., Lotfali E., Abolghasemi S., Mahmoudi S., Sadeghi F., Khalandi H., Ghasemi R., Shamsaei S. (2022). Anti-Biofilm Properties of Eucalyptol in Combination with Antifungals against *Candida albicans* Isolates in Patients with Hematological Malignancy. Arch. Microbiol..

[23] Al-Ghanayem A.A. (2022). Phytochemical Analysis of *Cymbopogon flexuosus* (Lemongrass) Oil, Its Antifungal Activity, and Role in Inhibiting Biofilm Formation in *Candida albicans* MTCC854. J. King Saud Univ. Sci..

[24] D’Arrigo M., Bisignano C., Irrera P., Smeriglio A., Zagami R., Trombetta D., Romeo O., Mandalari G. (2019). In Vitro Evaluation of the Activity of an Essential Oil from *Pistacia vera* L. Variety Bronte Hull against *Candida* Sp. BMC Complement. Altern. Med..

[25] Sahal G., Woerdenbag H.J., Hinrichs W.L.J., Visser A., Tepper P.G., Quax W.J., van der Mei H.C., Bilkay I.S. (2020). Antifungal and Biofilm Inhibitory Effect of *Cymbopogon citratus* (Lemongrass) Essential Oil on Biofilm Forming by *Candida tropicalis* Isolates; an in Vitro Study. J. Ethnopharmacol..

[26] Kojic E.M., Darouiche R.O. (2004). *Candida* Infections of Medical Devices. Clin. Microbiol. Rev..

[27] Mutlu-Ingok A., Devecioglu D., Dikmetas D.N., Karbancioglu-Guler F., Capanoglu E. (2020). Antibacterial, Antifungal, Antimycotoxigenic, and Antioxidant Activities of Essential Oils: An Updated Review. Molecules.

[28] Etxaniz A., González-Bullón D., Martín C., Ostolaza H. (2018). Membrane Repair Mechanisms against Permeabilization by Pore-Forming Toxins. Toxins.

[29] Liao S., Yang G., Huang S., Li B., Li A., Kan J. (2022). Chemical Composition of *Zanthoxylum schinifolium* Siebold & Zucc. Essential Oil and Evaluation of Its Antifungal Activity and Potential Modes of Action on *Malassezia restricta*. Ind. Crops Prod..

[30] Zore G.B., Thakre A.D., Rathod V., Karuppayil S.M. (2011). Evaluation of Anti-Candida Potential of Geranium Oil Constituents against Clinical Isolates of *Candida albicans* Differentially Sensitive to Fluconazole: Inhibition of Growth, Dimorphism and Sensitization. Mycoses.

[31] Ramage G., Milligan S., Lappin D.F., Sherry L., Sweeney P., Williams C., Bagg J., Culshaw S. (2012). Antifungal, Cytotoxic, and Immunomodulatory Properties of Tea Tree Oil and Its Derivative Components: Potential Role in Management of Oral Candidosis in Cancer Patients. Front. Microbiol..

[32] Ding Y., Zhang K., Yin Y., Wu J. (2022). D319 Induced Antifungal Effects through ROS-Mediated Apoptosis and Inhibited Isocitrate Lyase in *Candida albicans*. Biochim. Biophys. Acta Gen. Subj..

[33] Yang S.-K., Yusoff K., Thomas W., Akseer R., Alhosani M.S., Abushelaibi A., Lim S.-H.-E., Lai K.-S. (2020). Lavender Essential Oil Induces Oxidative Stress Which Modifies the Bacterial Membrane Permeability of Carbapenemase Producing *Klebsiella pneumoniae*. Sci. Rep..

[34] Perrone G.G., Tan S.-X., Dawes I.W. (2008). Reactive Oxygen Species and Yeast Apoptosis. Biochim. Biophys. Acta Mol. Cell Res..

[35] Čáp M., Váchová L., Palková Z. (2012). Reactive Oxygen Species in the Signaling and Adaptation of Multicellular Microbial Communities. Oxid. Med. Cell. Longev..

[36] Maurya I.K., Pathak S., Sharma M., Sanwal H., Chaudhary P., Tupe S., Deshpande M., Chauhan V.S., Prasad R. (2011). Antifungal Activity of Novel Synthetic Peptides by Accumulation of Reactive Oxygen Species (ROS) and Disruption of Cell Wall against *Candida albicans*. Peptides.

[37] Souza P.F.N., Marques L.S.M., Oliveira J.T.A., Lima P.G., Dias L.P., Neto N.A.S., Lopes F.E.S., Sousa J.S., Silva A.F.B., Caneiro R.F. (2020). Synthetic Antimicrobial Peptides: From Choice of the Best Sequences to Action Mechanisms. Biochimie.

[38] Oliveira A.R.M.F., Jezler C.N., Oliveira R.A., Mielke M.S.E., Costa L.C.B. (2012). Determinação Do Tempo de Hidrodestilação e Do Horário de Colheita No Óleo Essencial de Menta. Hortic. Brasc.

[39] Clinical and Laboratory Standards Institute (2008). M27-A3: Reference Method for Broth Dilution Antifungal Susceptibility Testing of Yeasts.

[40] Oliveira J.T.A., Souza P.F.N., Vasconcelos I.M., Dias L.P., Martins T.F., Van Tilburg M.F., Guedes M.I.F., Sousa D.O.B. (2019). Mo-CBP3-PepI, Mo-CBP3-PepII, and Mo-CBP3-PepIII Are Synthetic Antimicrobial Peptides Active against Human Pathogens by Stimulating ROS Generation and Increasing Plasma Membrane Permeability. Biochimie.

[41] Dias L.P., Souza P.F.N., Oliveira J.T.A., Vasconcelos I.M., Araújo N.M.S., Tilburg M.F.V., Guedes M.I.F., Carneiro R.F., Lopes J.L.S., Sousa D.O.B. (2020). RcAlb-PepII, a Synthetic Small Peptide Bioinspired in the 2S Albumin from the Seed Cake of *Ricinus communis*, Is a Potent Antimicrobial Agent against *Klebsiella pneumoniae* and *Candida parapsilosis*. Biochim. Biophys. Acta Biomembr..

[42] Dikalov S.I., Harrison D.G. (2014). Methods for Detection of Mitochondrial and Cellular Reactive Oxygen Species. Antioxid. Redox Signal..

[43] Staniszewska M., Bondaryk M., Swoboda-Kopec E., Siennicka K., Sygitowicz G., Kurzatkowski W. (2013). Candida albicans Morphologies Revealed by Scanning Electron Microscopy Analysis. Braz. J. Microbiol..

